# Hypofibrinolysis in type 2 diabetes and its clinical implications: from mechanisms to pharmacological modulation

**DOI:** 10.1186/s12933-021-01372-w

**Published:** 2021-09-22

**Authors:** Agata Hanna Bryk-Wiązania, Anetta Undas

**Affiliations:** 1 Department of Endocrinology, Jagiellonian University Medical College, Kraków, Poland; 2 University Hospital, Kraków, Poland; 3 Institute of Cardiology, Jagiellonian University Medical College, 80 Prądnicka St., 31-202 Kraków, Poland; 4 John Paul II Hospital, Kraków , Poland

**Keywords:** Type 2 diabetes, Fibrinolysis, Cardiovascular mortality

## Abstract

A prothrombotic state is a typical feature of type 2 diabetes mellitus (T2DM). Apart from increased platelet reactivity, endothelial dysfunction, hyperfibrinogenemia, and hypofibrinolysis are observed in T2DM. A variety of poorly elucidated mechanisms behind impaired fibrinolysis in this disease have been reported, indicating complex associations between platelet activation, fibrin formation and clot structure, and fibrinolysis inhibitors, in particular, elevated plasminogen antigen inhibitor-1 levels which are closely associated with obesity. Abnormal fibrin clot structure is of paramount importance for relative resistance to plasmin-mediated lysis in T2DM. Enhanced thrombin generation, a proinflammatory state, increased release of neutrophil extracellular traps, elevated complement C3, along with posttranslational modifications of fibrinogen and plasminogen have been regarded to contribute to altered clot structure and impaired fibrinolysis in T2DM. Antidiabetic agents such as metformin and insulin, as well as antithrombotic agents, including anticoagulants, have been reported to improve fibrin properties and accelerate fibrinolysis in T2DM. Notably, recent evidence shows that hypofibrinolysis, assessed in plasma-based assays, has a predictive value in terms of cardiovascular events and cardiovascular mortality in T2DM patients. This review presents the current data on the mechanisms underlying arterial and venous thrombotic complications in T2DM patients, with an emphasis on hypofibrinolysis and its impact on clinical outcomes. We also discuss potential modulators of fibrinolysis in the search for optimal therapy in diabetic patients.

## Introduction

It is estimated that diabetes mellitus (DM) had a global prevalence of 9.3% in 2019, of which 90% was accounted for by type 2 diabetes mellitus (T2DM) [1]. The typical patient with T2DM is obese, male, aged 63 years, suffering from T2DM for 10 years [2], a smoker, treated with antihypertensives, statin and metformin, with a glycated hemoglobin (HbA1c) of 55 mmol/mol (7.2%) [3]. Additionally, worse glycemic control is seen in patients with a positive family history of T2DM [4]. The prevalence of cardiovascular disease (CVD) among patients with T2DM varies from 21 [5] to 32% [2, 3], which is much higher than in the general population (10.6%) [6]. Coronary heart disease (CHD) has a prevalence of 21.2% in this patient population [2], corresponding to an age-standardized incidence rate of CHD of 28.8 and 23.3 per 1000 person-years for male and female T2DM patients, respectively [7]. Type 2 diabetes mellitus confers a two-fold increased risk of ischemic stroke [8] and a three–fivefold increased risk of myocardial infarction (MI) [9], especially among patients with insufficient control of cardiovascular risk factors [10]. In contrast to the well-established association of T2DM with arterial thromboembolism, its link with venous thromboembolism (VTE) is controversial [11], with a comparable age-adjusted incidence rate of VTE events in DM and the general population of 2.12 [12] vs. 1.83 [11] per 1000 person-years, respectively. The most common manifestation of VTE among diabetic patients is deep vein thrombosis (DVT, 72%) [13]. Diabetes mellitus has been reported to increase the risk of VTE by 60%, but only before adjustment for body-mass index (BMI) [12], hospitalization, major surgery, medical illness, or nursing home confinement [14]. Although the prevailing view is that T2DM is not an independent risk factor for VTE [15], it can be considered as a common comorbidity occurring in 19.1% of T2DM patients and is associated with a 74% increased risk for recurrent DVT, 40% increase in major bleeding in patients receiving anticoagulation [13], as well as a 2.3-fold increase in post-thrombotic ulcers [16]. Taken together, the current evidence indicates that T2DM substantially elevates arterial thromboembolic risk, in particular MI and stroke, while its impact on VTE risk and complications is largely associated with increased BMI.

## Clinical significance of hypofibrinolysis in T2DM

There are several assays used to asses fibrinolysis, some of them, like thromboelastography, might be used in a personalized medicine approach treatment regimes, as presented [17–20] in Table 1. Impaired plasmin-mediated fibrinolysis in T2DM has been convincingly demonstrated by several groups [21–23]. The clinical features of a T2DM patient with the worst fibrinolytic profile include women with an increased waist-hip circumference and decreased high-density-lipoprotein cholesterol, and who were diagnosed with T2DM at least 5 years earlier [23, 24]. Female sex and T2DM duration exceeding 5 years have been associated with an 8.9 and 9.7% increased lysis time, respectively [23, 24]. If the genotyping data were available and a patient was included in the 3% of T2DM patients being carriers of the BβArg448Lys variant in two alleles, the turbidity lysis time would be increased by 23.3% when compared to non-carriers and by 17% when compared to carriers of one allele [25]. If a T2DM patient was diagnosed with advanced CHD requiring coronary artery bypass graft surgery, the lysis time would be increased by 5.4% when compared with non-T2DM patients [26]. Moreover, in patients with a history of MI, measurement of lysis time may help to further assess the cardiovascular risk. Analysis of 4354 patients following acute MI, enrolled in the PLATelet inhibition and patient Outcomes (PLATO) trial, has demonstrated increasing DM prevalence among each subsequent quartile of lysis time [27]. Indeed, a sub-study devoted to 974 patients with DM has documented each 50% increase in lysis time being associated with a 21% increase in cardiovascular death or spontaneous MI, while risk for cardiovascular death alone was even higher (36%) [28]. Such results were obtained when the model was adjusted for age, gender, BMI, smoking history, hypertension, dyslipidemia, chronic kidney disease, acute and previous MI, congestive heart failure, revascularization, ischemic stroke, peripheral artery disease, and antiplatelet treatment [28]. The plethora of adjustments allows to appreciate that hypofibrinolysis in T2DM is associated with cardiovascular death independent of traditional risk factors. Increased clot density in T2DM patients, investigated using another plasma-based assay, has been linked to a 5.4-fold increase in cardiovascular mortality in T2DM patients [29]. Similar as above, adjustment for CVD history prior to the study enrollment, nephropathy, or treatment with metformin did not influence the hazard ratios of cardiovascular death [29]. On the other hand, T2DM patient who suffered their first episode of VTE had a 17% increased turbidity clot lysis time when compared to control subjects [30]. In summary, turbidity lysis time may be useful in the characterization of current and future cardiovascular risk. Key genetic and environmental factors affecting fibrinolysis in T2DM patients are presented in Table 2.

**Table 1:** The most commonly used plasma-based global fibrinolysis assays

	Clot lysis time by Lisman (min) [17]	Turbidity lysis time by Carter (s) [18]	Clot lysis time by Pieters (min) [19]	Clot lysis time in TEG (min) [20]
Reagents/material	Plasma	Plasma	Plasma	Whole blood
Coagulation trigger	Tissue factor 6 pM	Thrombin 0.03 NIH U/mL	Thrombin 0.5 NIH U/mL	Kaolin (tissue factor may be used additionally)
Calcium chloride	17 mM	7.5 mM	15 mM	2 M
Tissue plasminogen activator	56 ng/mL	83 ng/mL	18 ng/mL	–
Phospholipids	10 μM	–	10 μM	–

The final concentration of a reagent was presented, TEG denotes thromboelastography

**Table 2:** Factors affecting fibrinolysis in patients with type 2 diabetes mellitus (T2DM)

Study	No. of T2DM/controls age, BMI	Method for assessing fibrinolysis	T2DM patients	Control subjects	Relative difference and p-value*	Factors affecting fibrinolysis
Genetic						
Greenhalgh et al. [25]	822/0 68 (60–75) yrs, 31.0 ± 5.4 kg/m^2^	Plasma-derived clots Turbidimetric assay	763 ± 322 s (carriers of Bβ448Lys) 719 ± 351 s (Bβ448Arg)	–	Carriers of Bβ448Lys vs. Bβ448Arg + 6.1% p = 0.01	Fibrinogen Bβ448Lys variant
	36/0	Purified-fibrinogen derived clots Turbidimetric assay	517 ± 65 s (Lys/Lys) 442 ± 87 s (Arg/Lys) 419 ± 64 s (Arg/Arg)		Lys/Lys vs. Arg/Arg + 23.3% p = 0.003 Lys/Lys vs. Arg/Lys + 17.0% p = 0.05	
Molecular/environmental						
Dunn et al. [21]	25/25 61 ± 11 yrs, 29.3 ± 6.1 kg/m^2^	Fibrin formed from purified fibrinogen tPA-induced lysis assessed in confocal microscope	1.35 ± 0.37 μm/min	2.92 ± 0.57 μm/min	− 53.8% p < 0.0001	Decreased plasmin generation Reduced equlibrium binding between tPA and Glu-plasminogen, and fibrin Increased cross-linkage of factor XIII to fibrin HbA1c Posttranslational modifications of fibrinogen
Meltzer et al. [30]	71(DM + VTE)/2389 (non-DM + VTE) 49 (19–71) yrs, n.d	Plasma-based Lisman method	81.1 [95% CI 54.2–140.7] min	69.3 [95% CI 49.3–102.5] min	+ 17% p-value not specified	First episode of VTE
Meltzer et al. [139]	22/620 57.4 yrs, n.d	Plasma-based Lisman method	74.0 [95% CI 55.9–133.1] min	77.7 [95% CI 57.7–108.0] min	− 4.8% not significant	First myocardial infarction
Alzahrani et al. [24]	875/0 68.2 (60–75) yrs, 20.3 ± 0.3 kg/m^2^	Plasma-based Turbidimetric method	803 ± 20 (female) 665 ± 12 (male)	–	Female vs. male + 20.8% p < 0.001	Female sex Younger age in male Greater WCF in women HbA1c in men Lower HDL-cholesterol in women Lower eGFR Smoking in men Ischemic heart disease in men PAI-1
Bochenek et al. [26]	67(T2DM + CAD)/67 (non-T2DM + CAD) 65.6 ± 7.8 yrs, 29.6 ± 4.0 kg/m^2^	Plasma-based Turbidity	9.42 ± 1.47 min	8.94 ± 1.23 min	+ 5.4% p = 0.04	P-selectin vWF PAI-1 Fibrinogen
Neergard-Petersen et al. [96]	148 (T2D + CAD)/433 (non-T2DM + CAD) 65 ± 8 yrs, 30 ± 5 kg/m^2^	Plasma-based Turbidimetric method	804 (618; 1002) s	750 (624; 906) s	+ 7.2% p = 0.03	Quantitative rather than qualitative changes in fibrinogen CRP, complement C3, interleukin-6 Female sex BMI
		Purified-fibrinogen Turbidimetric method	605 ± 163 s	490 ± 99 s	+ 23.5% p = 0.21	
Hess et al. [85]	837/0 67.9 ± 4.2 yrs, 30.7 (27.3–34.4) kg/m^2^	Plasma-based Turbidimetric	618 (480–816) s	–	–	Complement C3 PAI-1
Konieczynska et al. [23]	156/0 66 (60–73) yrs, 32 ± 5.4 kg/m^2^	Plasma-based William’s method	10.2 ± 0.1 min (T2DM > 5 years) 9.3 ± 0.1 min (T2DM ≤ 5 years)	–	T2DM > 5 years vs. T2DM ≤ 5 years + 9.7% p < 0.0001	Time since T2DM diagnosis > 5 years HbA1c > 6.5% Fibrinogen PAI-1 antigen Peak thrombin
		Plasma-based Lisman method	101.5 ± 1.8 min (T2DM > 5 years) 89.7 ± 1.6 min (T2DM ≤ 5 years)		T2DM > 5 years vs. T2DM ≤ 5 years + 13.2% p < 0.0001	
Lados-Krupa et al. [100]	163/0 65 ± 8.8 yrs, 31.9 ± 5.2 kg/m^2^	Plasma-based Tissue factor and tPA	95.9 [95% CI: 91.0–100] min (Hba1c > 7%) 94.7 [95% CI: 91.5–97.9] min (Hba1c ≤ 7%)	–	Hba1c > 7% vs. Hba1c ≤ 7% + 1.3% p = 0.069	Oxidized LDL-cholesterol
Gajos et al. [75]	165/0 Data available for subgroups	Plasma-based Thrombin and tPA	10.49 ± 0.97 min (low glucose, < 4.5 mmol/l) 9.55 ± 0.91 min (medium glucose, 4.5–6.0 mmol/l) 9.79 ± 1.11 min (high glucose, > 6.0 mmol/l)	–	Medium glucose vs. low − 9.0% p < 0.05	Glucose < 4.5 mmol/l
					High glucose vs. low − 6.7% p < 0.05	
Bryk et al. [58]	113/0 63.8 ± 8.2 yrs, 32 (29.4–37.2) kg/m^2^	Plasma-based Lisman method	114.0 (99.3–126.8) min (H3Cit ≥ 7.36 ng/ml) 87.0 (78.3–100.0) min (H3Cit < 7.36 ng/ml)	–	H3Cit ≥ 7.36 ng/ml vs. H3Cit < 7.36 ng/ml + 31.0% p < 0.001	H3Cit cfDNA PAI-1 CVD
Bryk et al. [89]	113/0 63.8 ± 8.2 years, 32 (29.4–37.2) kg/m^2^	Plasma-based Turbidity	471 (401–555) s (α2-antiplasmin incorporation ≥ 29.79 mg/dl) vs. 383 (345–435) s (α2-antiplasmin incorporation < 29.79 mg/dl)	–	α2-antiplasmin incorporation ≥ 29.79 mg/dl vs. α2-antiplasmin incorporation < 29.79 mg/dl + 23.0% p < 0.001	α2-antiplasmin incorporation Fibrinogen Female gender PAI-1 BMI
Pharmacological						
Grant [140]	25/23 n.d., > 25 kg/m^2^	Euglobulin clot lysis time	50.9 ± 98.9 min (mean change from baseline after 12 wks of treatment with 3 g metformin)	n.d	Change from baseline after 12 wks of treatment with 3 g metformin no baseline data p = 0.026	HbA1c, insulin, glucose, triglycerides, cholesterol PAI-1, tPA
			60.6 ± 84.7 min (mean change from baseline after 6 months of treatment with 1.5 g metformin)		Change from baseline after 6 months of treatment with 1.5 g metformin no baseline data p = 0.012	
Pieters et al. [97]	7/5 53 (49.1–56.9) yrs, n.d	Fibrin formed from purified fibrinogen tPA-induced lysis assessed in confocal microscope	3.08 [2.48;3.25] μm/min (at baseline)	8.52 [6.18; 8.59] μm/min	T2DM at baseline vs. control at baseline − 63.8% p = 0.06	Glycemic control Fibrinogen glycation
			3.27 [2.92;3.72] μm/min (after treatment with insulin)	8.21 [6.50; 8.64] μm/min	Baseline T2DM vs. T2DM after treatment with insulin -5.8% p = 0.02	
Bryk et al. [99]	7/0 62 (60–63) yrs, n.d	Plasma-based Pieters method	130.0 (117.8–233.5) min (at baseline) 127.5 (125.0–262.0) min (after 1-month treatment with 75 mg aspirin once daily)	–	Baseline vs. after treatment with aspirin + 2.0% p = 1.0	Fibrinogen glycation and acetylation sites

Numerical data were presented as mean ± standard deviation, or median (interquartile range) or median [95% confidence interval, CI]. Relative difference and p-value have been plotted between T2DM patients and control groups, unless stated otherwise

*BMI* body-mass-index, *CAD* coronary artery disease, *cfDNA* cell-free DNA, *CRP* C-reactive protein, *CVD* cardiovascular disease, *eGFR* estimated glomerular filtration rate, *HbA1c* glycated hemoglobin, *H3Cit* citrullinated histone H3, *HDL* high-density lipoprotein, *LDL* low-density lipoprotein, *n.d.* no data, *PAI-1* plasminogen-activator inhibitor, *tPA* tissue plasminogen activator, *VTE* venous thrombomebolism, *vWF* von Willebrand factor, *WCF* waist circumference

## Summary of the literature

The most relevant research on hypofibrinolysis and its clinical implications in T2DM was reviewed. We included papers regarding the cellular components, including platelet hyperactivity and pathological structure and function of erythrocytes. They have been followed by papers describing the endothelial dysfunction, enhanced thrombin generation (TG) and inflammatory state, including neutrophil cellular traps (NETs). Then we proceeded to the review of literature in the topic of qualitative and quantitative changes of fibrinogen, with a special emphasis on the posttranslational modifications of fibrinogen. This has been followed by the analogical modifications of proteins involved in fibrinolysis. In the part devoted to the pharmacological interventions, we presented data on antidiabetic drugs, aspirin and FXa-inhibitors. Results of basic research and clinical trials were selected from PubMed and Web of Science from January 2000 to May 2021, supported by a few seminal papers from previous years.

## Mechanisms of hypofibrinolysis in T2DM

**Fig. 1:**
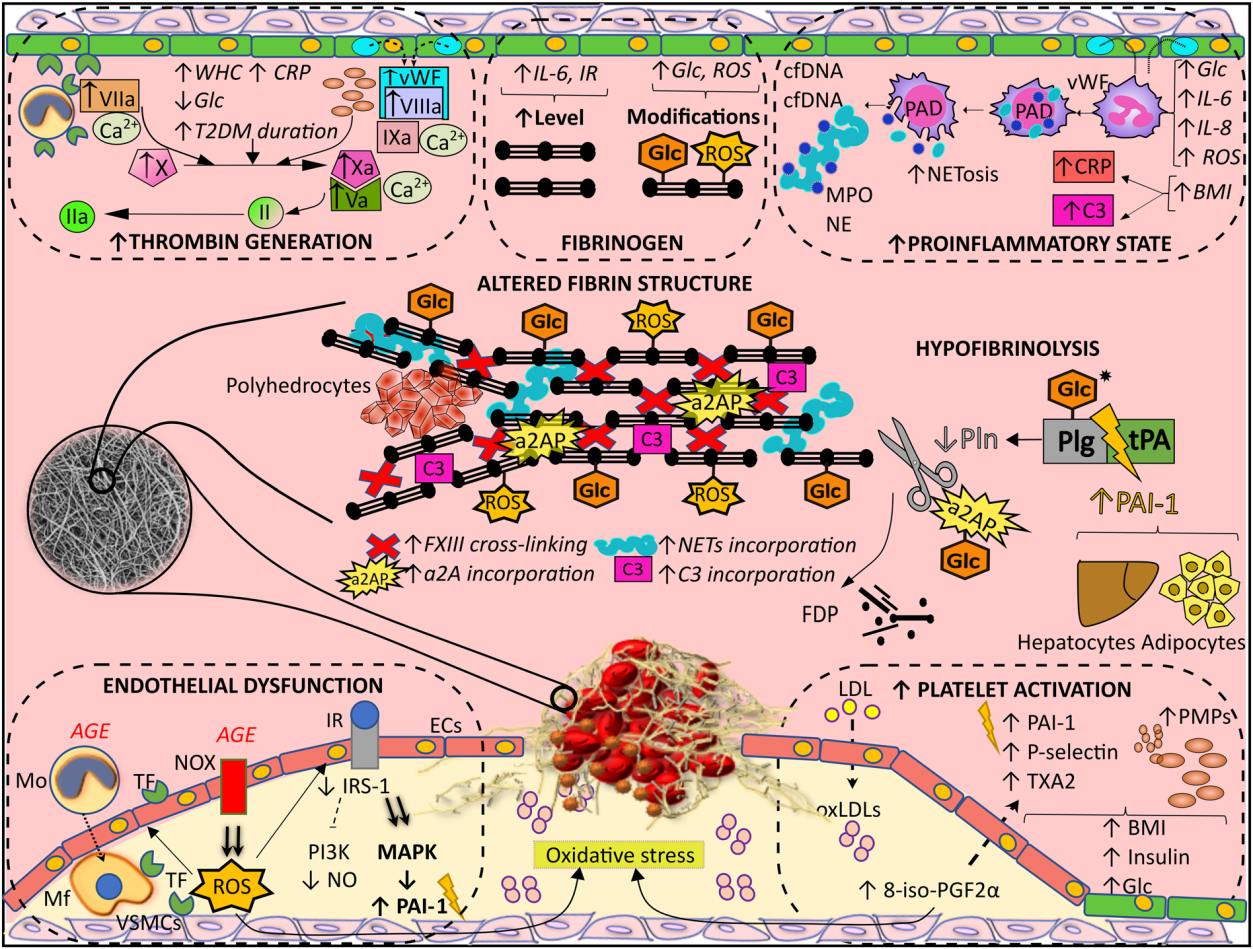
Mechanisms involved in hypofibrinolysis in type 2 diabetes mellitus (T2DM) patients. The main contributors to hypofibrinolysis in T2DM are platelet activation, endothelial cells (ECs) dysfunction, enhanced thrombin generation, proinflammatory state, increased fibrinogen level along with its modifications, and altered fibrin structure. Obesity represented by high body mass index (BMI), hyperinsulinemia, and hyperglycemia (high glucose, Glc) all lead to platelet activation reflected by increased release of thromboxane A2 (TXA2), P-selectin, plasminogen activator inhibitor 1 (PAI-1), and platelet microparticles (PMPs). Another contributor to platelet activation is oxidative stress, which is reflected by increased synthesis of F2-isoprostane 8-iso-prostaglandin F_2α_ (8-iso-PGF_2α_), a product of low-density-lipoprotein (LDL) cholesterol peroxidation, represented by oxidized LDL (oxLDL). Down-regulation of the insulin receptor substrate 1 (IRS-1) and phosphatidylinositol 3-kinase (PI3K) pathways with enhancement of mitogen-activated protein kinase (MAPK) result in decreased nitric oxide (NO) synthesis and increased PAI-1 release, leading to endothelial dysfunction. Advanced glycation end-products (AGE) stimulate overactive NADPH oxidases (NOX), reactive oxygen species (ROS)-producing enzyme complexes, which in turn generates tissue factor (TF) in ECs. AGE stimulate monocytes (Mo)/macrophages (Ma) to produce increased amounts of TF. Another source of TF are the vascular smooth muscle cells (VSMCs). Increased TF initiates the extrinsic pathway of coagulation and together with factor VII (VIIa, activated factor VII) lead to enhanced thrombin generation. Weibel-Palade bodies release increased amounts of von Willebrand factor (vWF), which along with increased factor VIII (VIIIa, activated factor VIII) and factor IX (IXa, activated factor IX), form the intrinsic pathway of thrombin generation. Increase in both components of the prothrombinase complex, activated factor X (Xa) and V (Va). Other factors underlying enhanced thrombin generation are central obesity represented by increased waist-hip circumference (WHC) ratio, elevated C-reactive protein (CRP), low glycemia, and time since T2DM diagnosis exceeding 5 years. Hyperglycemia, increased interleukins 6 and 8 (IL-6 and IL-8), along with ROS stimulate neutrophils to form the neutrophil extracellular traps (NETs), with an important stage of chromatin decondensation mediated by peptidylarginine deiminase 4 (PAD4), followed by a release of nuclear components depicted in light blue, i.e., cell-free DNA (cfDNA), citrullinated histone H3 (H3Cit), and granular components depicted in dark blue, i.e., myeloperoxidase (MPO) and neutrophil elastase (NE). Obesity represented by increased BMI elevates both CRP and complement C3 (C3) levels. IL-6 and insulin resistance contribute to elevated fibrinogen concentration, while hyperglycemia and ROS result in posttranslational modifications, such as fibrinogen glycation (–Glc) and oxidation (–ROS). The fibrin network formed from modified fibrinogen, with increased amounts of incorporated α2-antiplasmin (a2AP), NETs, and complement C3, characterized by enhanced crosslinking by factor XIII (FXIII), dense and less permeable, being composed of thinner and highly branched fibrin fibers. Additionally, increased amount of polyhedrocytes are found in the contracted thrombi of diabetic patients. Decreased plasmin generation, diminished binding of plasminogen and tissue plasminogen activator (tPA) to fibrin, along with increased PAI-1, originating from adipocytes and hepatocytes, are also involved in hypofibrinolysis observed in T2DM. Glycation of plasminogen was reported in type 1 diabetes mellitus patients and therefore is marked with asterisks. α2-antiplasmin is another protein implicated in fibrinolysis and found to be glycated in T2DM. *FDP,* fibrin degradation products

Key steps of fibrinolysis involve initiation and propagation [31]. Fibrinolysis starts with the colocation of plasminogen and tissue plasminogen activator (tPA) on fibrin, where they form a complex that stimulate generation of plasmin [31]. Plasmin cleaves fibrin producing fibrin degradation products and terminal C-lysines on fibrin providing additional positive feedback through enhanced plasminogen binding [31]. The two most critical serpin inhibitors in fibrinolysis are plasminogen activator inhibitor 1 (PAI-1) and α2-antiplasmin inhibitor [31]. The interaction between serpin inhibitors and target enzymes are modulated by fibrin and fibrinogen [31]. Multiple interacting mechanisms have been postulated to be involved in hypofibrinolysis in T2DM (Fig. 1).

### Platelet hyperreactivity

Studies spanning over more than 30 years have documented platelet hyperreactivity in T2DM, which is reflected by enhanced biosynthesis of thromboxane (TX) A2 [32, 33]. A study by Patrono et al. demonstrated that oxidative stress, reflected by a two-fold increased synthesis of F2-isoprostane 8-iso-prostaglandin F_2α_ (8-iso-PGF_2α_), a bioactive product of arachidonic acid peroxidation, linearly correlated with blood glucose levels, largely contributing to persistent platelet activation in T2DM [34]. The Italian investigators also found that postprandial hyperglycemia was associated with enhanced lipid peroxidation and platelet activation induced by increased production of TXA2 in newly diagnosed T2DM patients, with their subsequent reduction following acarbose treatment [35]. They also reported that 11-dehydro-TXB2 levels and 8-iso-PGF_2α_ excretion rates predicted soluble CD40 ligand (sCD40L) levels in T2DM patients in association with an inflammatory state, which was reflected by elevated C-reactive protein (CRP) concentrations [36]. Of note, a randomized controlled trial by Simeone et al. showed that weight loss, achieved through either lifestyle changes or an incretin-based therapy, was associated with a significant reduction in lipid peroxidation and TX-mediated platelet hyperreactivity [37]. Increased oxidative stress in subjects with T2DM is a consequence of metabolic abnormalities, including insulin resistance, hyperinsulinemia, hyperglycemia, and dyslipidemia [38], with obesity having a crucial role as a clustering factor for all of the above-mentioned abnormalities, leading to platelet dysfunction. Using a euglycemic-hyperinsulinemic clamp in lean non-diabetic subjects, Westerbacka et al. observed an inhibition of platelet deposition to collagen and a decrease in platelet aggregation to several agonists [39]. Inhibitory effects of insulin under similar conditions were not seen in obese patients [39]. This was confirmed during a sub-study of the Bypass Angioplasty Revascularization Investigation 2 Diabetes (BARI 2D) study, which demonstrated a positive correlation between BMI and adenosine diphosphate-stimulated increase in platelet-surface expression of P-selectin among 193 patients with T2DM and stable CHD [40]. Platelet hyperreactivity might contribute to hypofibrinolysis in T2DM in at least two ways. More than 90% of PAI-1 is stored in platelet α-granules but its substantial portion is inactive [41]. It has been reported that platelets of T2DM patients contain a decreased amount of PAI-antigen, while PAI-1 concentration and activity in plasma of T2DM patients are significantly higher than in controls [42]. An altered platelet membrane ultrastructure with apoptotic morphology and membrane has been reported in diabetic patents [43], along with enhanced platelet-derived microparticle (PMP) formation [44]. It has been recently demonstrated that under chronic hyperglycemia, protease-activated receptor 4 promotes release of PMPs through a Ca^2+^ -calpain dependent mechanism [45]. Fibrin clots formed in the presence of microparticles have been shown to be less susceptible to tPA-induced fibrinolysis in healthy subjects [46], and this can be most likely extrapolated to diabetic patients. In summary, enhanced TX-dependent platelet activation is a typical phenomenon in T2DM patients and is associated with oxidative stress, a proinflammatory state, hyperglycemia, and insulin resistance, which may contribute to hypofibrinolysis, at least in part through enhanced release of PAI-1 and PMP.

### Pathological structure and function of erythrocytes

Recent evidence suggests that erythrocytes are implicated into hypofibrinolysis in T2DM patients. As a consequence of a decrease in the cholesterol to phospholipid ratio, increased membrane lipid peroxidation, and glycosylation of cytoskeletal proteins, the membrane of red blood cells becomes rigid and non-deformable in diabetic subjects [47]. It is known that erythrocytes in contracted thrombi form close-packed polyhedral erythrocytes, polihedrocytes, which contribute to increased resistance to fibrinolysis [48]. Enhanced formation of polihedrocytes have been reported in T2DM, and increased fasting glucose and glycated hemoglobin have been correlated with content of polyhedral erythrocytes [49]. It has been suggested that the decreased lysability of such clots results from decreased penetration of lytic enzymes into tightly packed clots rich in polyhedrocytes [48]. Indeed, diabetic patients who formed a large amounts of polyhedrocytes within thrombi have been demonstrated to have longer clot lysis time when compared to those with lower amounts of polyhedrocytes [49]. In conclusion, changes in ultrastructure of the erythrocyte membrane result in its altered mechanical properties, which might impair fibrinolysis in T2DM.

### Endothelial dysfunction

Decreased bioavailability of nitric oxide (NO) is a typical feature of endothelial dysfunction in T2DM, leading to vasoconstriction [50]. It is unclear whether decreased NO directly contributes to hypofibrinolysis. More importantly, decreased bioavailability of NO is mediated by the same molecular pathway as increased expression of PAI-1, i.e., a down-regulation of insulin receptor substrate 1 in endothelial cells with a consequent shift from the phosphatidylinositol 3-kinase pathway to mitogen-activated protein kinase signaling [51]. Several recent studies [41] have shown PAI-1 levels to be elevated in T2DM patients by approximately 25–280% when compared to nondiabetic subjects. Interestingly, hyperglycemia is not a required factor, while increased PAI-1 has also been reported during diabetic hypoglycemia [52, 53]. Endothelial release of PAI-1 is likely to partially account for this increase in PAI-1 measured in the blood of subjects with insulin-resistant states [54]. Apart from platelets, other sources of PAI-1 include hepatocytes [55] and adipocytes [56], with the latter being a proxy for a positive correlation between BMI and PAI-1 activity [57]. In a cross-sectional study of mostly obese patients (76%, median BMI 32 kg/m^2^), circulating PAI-1 antigen contributed in 13.8% to variance of clot lysis time determined using the assay of Lisman [58]. Data about thrombin-activatable fibrinolysis inhibitor (TAFI) in T2DM are rather limited, although there has been a report demonstrating that both antigen and activity are increased by approximately 25% and 75%, respectively [59]. In a cross-sectional study of 55 T2DM patients, soluble thrombomodulin, another marker of endothelial dysfunction and potent activator of TAFI, was reported to be increased by 45% in patients with an HbA1c of 9.1%, when compared to control subjects [60]. Moreover, T2DM is characterized by overactive NADPH oxidases (NOX), which are the source of reactive oxygen species (ROS) [61]. When they are activated by advanced glycation end-products (AGE), generation of tissue factor (TF) in human endothelial cells is initiated [62]. The main sources of TF, a key initiator of blood coagulation in vivo, are monocytes/macrophages and vascular smooth muscle cells [63]. Monocytes incubated in vitro with glycated albumin [64] or AGE [65] have been reported to express increased amounts of TF on the surface [64, 65], which is in line with data showing increased TF expression on monocytes isolated from T2DM patients when compared to healthy volunteers [65]. Type 2 diabetes mellitus patients were shown to have an approximately fourfold greater procoagulant activity of TF, which increased by 30% under hyperinsulinemic conditions and by 80% under the mutual action of hyperinsulinemia and hyperglycemia [66]. Tissue factor expression is regulated at the post-transcriptional level, being reduced by endothelial microRNA (miR)-19a and miR-181b, while increased by miR-126 [67, 68]. In summary, the current evidence convincingly shows that endothelial dysfunction in T2DM, driven by disturbed signaling in the arterial wall along with overactive NOX, is related to hypofibrinolysis through increased expression of PAI-1, TF, thrombomodulin, and most probably TAFI.

### Enhanced TG

Thrombin concentration influences both the fiber thickness and density of a fibrin clot [69]. High thrombin concentrations have been associated with relatively non-turbid, less permeable clots, with a dense network composed of relatively thin fibrin fibers [69]. Enhanced TG was demonstrated in a study involving a representative group of 60 T2DM patients at a typical age of T2DM diagnosis, namely 64 years, diagnosed with T2DM approximately 9 years prior, treated mainly with oral hypoglycemic drugs [70]. Such T2DM patients have been demonstrated to have a 14–25% decreased lag-time, 16–23% shorter time-to-peak, 15–45% increased peak thrombin concentration, and 15–28% increased endogenous thrombin potential (depending on the coagulation trigger and presence of thrombomodulin) when compared to control subjects [70]. Type 2 diabetes mellitus coexisting with CVD was associated with a 12% increase in endogenous thrombin potential, determined using calibrated automated thrombogram (CAT), when compared to diabetic patients without CVD [71]. Recently, our group has reported that among 133 patients with T2DM, increased peak thrombin concentration measured at baseline using CAT (> 283.5 nM) conferred a 5.6-fold greater risk of cardiovascular mortality during a 6-year follow-up [29]. Enhanced TG in T2DM has a multifactorial background, given the fact that platelet hyperreactivity and increased TF expression, along with endothelial injury, all lead to augmented factor Xa-mediated conversion of prothrombin to thrombin, the key enzyme in blood coagulation. Other postulated mechanisms leading to enhanced TG include increased cell-derived microparticles [70], increased coagulation factors (II, V, VII, VIII, and X) along with low levels of anticoagulants (protein C) [72], increased von Willebrand factor [73], high waist circumference, and a proinflammatory state reflected by increased CRP [74]. Recent studies have shown that low glycemia (< 4.5 mmol; + 16% higher peak thrombin vs. patients with glucose > 6 mmol/l) [75] and time since T2DM diagnosis > 5 years (+ 16% higher peak thrombin vs. patients with the T2DM duration of ≤ 5 years) contribute to enhanced thrombin generation [23]. In summary, current evidence supports the role of enhanced TG in the development of cardiovascular complications in T2DM patients, with some of these complications likely being related to hypofibrinolysis.

### Inflammatory state

Current data supports the view that DM is characterized by increased inflammation and by increased levels of CRP, Toll-like receptors 2 and 4, interleukins, and NOX [76]. In response to interleukins and ROS [77, 78], NETs are released from activated neutrophils. Neutrophil extracellular traps may promote thrombosis via several pathways. They provide the scaffold for thrombus formation, stimulate platelet adhesion and aggregation, bind fibrinogen [79], stimulate TG [80], and enhance the formation of thicker, more stable and rigid fibrin fibers displaying increased lysis time [81]. Although one study reported a weaker ability of neutrophils from T2DM patients to form NETs [78], the prevailing view is that hyperglycemia enhances NETosis, which is reflected by a 5.6-fold greater formation of NETs when neutrophils are incubated with 25 mM glucose [82]. In line with the latter concept, increased circulating markers of NETs (nucleosomes, extracellular DNA, and neutrophil elastase) have been found in the plasma of T2DM patients when compared to non-diabetic individuals [82]. Moreover, elevated markers of NETs formation have been reported in T2DM patients with HbA1c ≥ 8% or with concomitant CVD [58]. Growing evidence supports the view that formation of NETs is enhanced in T2DM patients and may contribute to the elevated risk for thromboembolic events in this disease [83]. Most importantly, elevated circulating markers of NETs, defined as citrullinated histone H3 and cell-free DNA in the top quartiles, have been found to predict clot lysis time when assessed by the Lisman method in T2DM patients, accompanied by CVD and other important modulators of fibrinolysis such as PAI-1 [58]. An increase in circulating markers of NETs is related to the level of glycemic control and interleukin 6 (IL-6)-mediated inflammatory state [58]. Using a proteomic approach, several proteins related to inflammatory processes have been identified in the plasma fibrin clot, including complement C3 [84]. Further studies have allowed to fully characterize the role of complement C3 in the clots of T2DM patients and have demonstrated the positive relationship between its incorporation into clots and BMI [85]. In contrast to CRP levels, complement C3 contributes to turbidimetric clot lysis to a similar extent as PAI-1[85]. Taken together, many recent investigations have revealed that NETs and complement C3 are novel contributors to hypofibrinolysis in T2DM. Their prognostic value regarding cardiovascular mortality in T2DM remains to be established.

### Hyperfibrinogenemia

Persistently increased plasma fibrinogen concentrations are a typical laboratory abnormality observed among T2DM patients, which is a consequence of increased IL-6 level and insulin resistance [9, 86]. Hypofibrinolysis seems to be driven by PAI-1 rather than by fibrinogen; however, fibrinogen concentrations may still contribute to hypofibrinolysis. It has been presented in plasma-purified models that increasing fibrinogen levels positively correlated with clot lysis [87] and also led to more rigid fibrin clots when assessed using the rheometric assay [88]. Among 502 patients, including 129 with metabolic syndrome, fibrinogen concentration contributed to variance in turbidimetric lysis assay parameters, explaining 8.6% of time from initiation of coagulation to the 50% clot lysis [18]. The contribution of PAI-1 to lysis parameters was comparable and ranged from 7 to 13% [18]. However, current evidence shows that the relationship between patient sex and impairment of fibrinolysis in T2DM appears to be independent of fibrinogen levels [24, 89]. Hyperfibrinogenemia and cardiovascular risk in T2DM have been studied by several investigators. In the Casale Monferrato Study, a fibrinogen concentration of > 4.1 g/l conferred a 61% increased risk for cardiovascular mortality during an 11-year follow-up of stable T2DM patients when compared to those with a fibrinogen concentration of < 3.0 g/l [90]. As shown in a subsequent study of nearly 3900 T2DM patients with CVD or at least one cardiovascular risk factor, the association between cardiovascular events and fibrinogen is attenuated when adjusted for IL-6 levels [91]. In a small observational study of 133 clinically stable T2DM patients, no difference was seen in the baseline fibrinogen concentration between patients who died from cardiovascular causes and survivors in the 6-year follow-up period [29]. Conversely, a greater baseline plasma fibrinogen concentration was noted in diabetic post-MI patients when compared to non-DM subjects [92]. Furthermore, a fibrinogen concentration of > 2.9 g/l conferred a 7.8-fold greater risk for a subsequent major cardiovascular event [92]. A meta-analysis published in 2013 involving over 33,000 patients, including 1000 with DM, showed that patients with or without DM did not differ regarding associations of fibrinogen with CVD and all-cause mortality [93]. Moreover, adding fibrinogen to the established risk factors did not improve the predictive accuracy [93]. Taken together, hyperfibrinogenemia might contribute to decreased fibrin clot lysability and subsequent thromboembolism in T2DM patients; however, its impact does not appear to be independent of other cardiovascular risk factors.

### Posttranslational modifications affecting fibrin clot structure

Abnormal fibrin clot structure in T2DM has been demonstrated in cross-sectional [23, 24, 94–96] and interventional studies [22, 97, 98], using assays based on purified fibrinogen [94, 97] or plasma [22–24, 95]. Because fibrin functions as both a cofactor and a substrate for the fibrinolytic enzyme plasmin, the fibrin structure influences the clot’s susceptibility to fibrinolysis [69]. A more rapid formation of dense, less permeable meshwork composed of thinner and highly branched fibers, observed in purified systems, has been attributed to mechanisms independent of increased fibrinogen levels. These mechanisms include modifications to the fibrinogen molecule, interference with fibrin polymerization, cross-linking by factor XIII (FXIII), and binding of tPA and plasminogen [94]. Three lysine residues on the α chain (K-539, K-208, K-448), previously identified to undergo glycation in mass-spectrometric analysis of plasma fibrin clots in T2DM patients, have been reported to be involved in the interaction with FXIII [99]. In 20 T2DM patients with an initial HbA1c of 11.7%, insulin treatment was associated with a 36% decrease in fibrinogen glycation, which corresponded to a 27% decreased rate of lateral aggregation and to 14% increased clot permeability [97]. However, fibrinogen glycation did not correlate with fibrin fiber extensibility, modulus, and stress relaxation [98]. Because the mass of glycated fibrinogen exceeded the sum of glucose and fibrinogen, subsequent modification of glycated fibrinogen by oxidation was postulated by Dunn et al. [94]. Indeed, markers of oxidative stress in plasma such as nitrotyrosine, soluble receptor for AGE, 8-iso-PGF2α, oxidized low-density lipoprotein, and total plasma carbonylation, have been correlated with plasma fibrin clot permeability [100, 101]. However, none of the residues responsible for binding plasmin to fibrin or for cleavage by plasmin [102] have been subjected to ozone-induced oxidation [103], although some of the oxidized sites were near these crucial sites. In summary, current evidence shows that the diabetic milieu promotes fibrinogen modifications, particularly glycations, which are of paramount importance in the formation of abnormal fibrin clot structure.

### Posttranslational modifications affecting proteins involved in fibrinolysis

In a purified system, clots from T2DM patients with an HbA1c of 7.9% have been demonstrated to have a twofold decreased clot lysis velocity along with 30% decreased binding of plasminogen and tPA to fibrin clots, as well as decreased plasmin activation [21]. Mass-spectrometry of the highly purified human fibrinogen (glycated in vitro) revealed that the lysine residues on the β chain (K-133) which underwent glycation were situated within the ‘‘plasmin-sensitive’’ coiled–coil region, near the sites of plasmin proteolysis [104]. Another glycation site on the fibrinogen α chain, which was previously implicated in the interaction with plasmin, has been identified from plasma-derived fibrin clots of T2DM patients [99]. Posttranslational modifications of plasminogen have been investigated in type 1 DM. Because hyperglycemia is the common core metabolic abnormality, results from these studies can most likely be extrapolated to T2DM patients. Two glycation sites involved in fibrin binding and plasminogen cleavage have been identified on plasminogen purified from 20 patients with type 1 DM. These patients had a mean age of 22 years, were 50% male, diagnosed with DM 11 years prior, treated with insulin [105]. Plasminogen purified from these patients showed 2.3-fold longer lysis time and 2.2-fold decreased catalytic efficiency [105]. In vitro, after incubation of α2-antiplasmin with glucose at concentrations encountered in T2DM, eleven glycation sites have been identified on α2-antiplasmin, including on four (K-418, K-427, K-434, K-441) out of six lysine residues, known to be important for mediating the interaction with plasmin [106], the biological significance of these glycations remains to be established. Interestingly, α2-antiplasmin incubation with glucose in similar conditions as above was associated with decreased α2-antiplasmin binding affinity to fibrin [107]. This finding seems to be counterintuitive to previous reports showing increased FXIII-induced cross-linkage of α2-antiplasmin to fibrin when compared to control subjects both in fibrin from fibrinogen purified from T2DM patients (by 20%) [21] and in a plasma-based assay (by 6%) [108]. It appears that the results strongly depend on the model chosen for investigation, with plasma-based assays probably being the most appropriate. Finally, increased α2-antiplasmin incorporation into fibrin has been implicated in compromised fibrinolysis in women with T2DM when compared to men with T2DM [24]. Perhaps, this could be due to different glycation patterns; however, this should be investigated in future studies. In summary, modifications of both fibrinogen and plasminogen are implicated in hypofibrinolysis in T2DM. Moreover, α2-antiplasmin undergoes glycation, with possible links to compromised fibrinolysis, which requires further studies.

### Iron metabolism and fibrinolysis

Individuals with T2DM have higher plasma levels of ferritin and tend to have higher total plasma levels of iron [109]. Trivalent iron, that circulates in blood, initiates a hydroxyl radical-catalyzed conversion of fibrinogen into a fibrin-like polymer (parafibrin) that is resistant to the proteolytic dissolution and causes chronic inflammation [110]. Interestingly, very recently it has been shown that empagliflozin increases erythropoiesis and augments iron utilization [111]. Whether this would serve some pathway how empagliflozin could decrease prothrombotic tendency in T2DM, remains to be established. Taken together, current data suggest that iron may be involved in hypofibrinolysis in T2DM.

## Pharmacological interventions

### Antidiabetic drugs

Metformin has an established role in the prevention of T2DM-related death, reducing it by 42% when compared to dietary treatment alone [112] and by 36% when compared to sulfonylureas [113]. Metformin has been demonstrated to decrease platelet activity and concentrations of fibrinogen, PAI-1, and tPA [86, 114]; to reduce oxidative stress as reflected by levels of 8-iso-PGF2α, methylglyoxal, and carboxymethyl-lysine [115, 116]; to decrease the formation of NETs, and levels of circulating cell-free DNA and IL-6 [117]; and to improve endothelial function [118]. Metformin exerts vasoprotective effect by reducing the TF activity in the blood of T2DM patients and inhibiting TF transcription via AMP-activated protein kinase in human monocytes [119]. In pooled diabetic plasma-clot turbidity, metformin increased the lag period and decreased the maximum turbidity, along with decreasing FXIII antigen level and activity. Additionally, metformin was shown to inhibit fibrinopeptide cleavage from fibrinogen [120]. The effect of insulin on the fibrin clot structure in T2DM has been considered controversial, being absent in plasma-based clot assays with high fibrinogen levels in T2DM and control patients [22] but present in purified-fibrinogen assays where insulin decreased lateral aggregation and increased permeability and lysis rate [97].

Data regarding the effects of new antidiabetic drugs on fibrinolysis are rather scarce. In vitro, dipeptidyl peptidase-4 (DPP-4) has been demonstrated to increase ROS generation and expression of the receptor for AGE in endothelial cells. Linagliptin, a DPP-4 inhibitor, has been shown to prevent these unfavorable effects, along with decreasing PAI-1 gene expression in endothelial cells [121]. Additionally, both vildagliptin and sitagliptin have been reported to decrease PAI-1 [122, 123] levels in T2DM patients, in contrast to glucagon-like peptide-1 receptor agonists [124]. In a randomized trial, a 12-months long treatment with empagliflozin (10 mg/day, n = 31) has resulted in 25% decrease in PAI-1 concentration when compared with standard therapy [125]. This has been however attributed to the synergistic action on glucose metabolism, the weight loss and changes in leptin concentrations [125]. In polycystic ovary syndrome, exenatide was shown to decrease lysis time, without having any effect on clot density [124]. However, to date, there have been no reports on the effect of the new antidiabetic drugs on clot lysis time in patients with T2DM.

### Aspirin and FXa-inhibitors

The antiplatelet effects of aspirin in T2DM patients were extensively explored in a study by Patrono et al., which showed that sCD40L and 11-dehydro-TXB2 levels decreased after 7 days of aspirin use (30, 100 or 325 mg daily) [36]. Sub-optimal platelet inhibition in approximately one-third of T2DM patients on a once-daily low-dose aspirin regimen has been represented by the shorter-than-expected inhibition of platelet-derived TXA2, supporting the concept of accelerated cyclooxygenase-1 renewal during the dosing interval [126]. The main drivers of this reduced responsiveness appeared to be obesity and increased platelet turnover, which is the reason why a twice-daily aspirin regimen or doubling the once-daily dose have been suggested in patients with a BMI > 35 kg/m^2^ [127]. A recent large meta-analysis failed to show any effect of aspirin 81–650 mg daily in the primary prevention of cardiovascular events in diabetic patients [128]. Although DM is an indication for initiating aspirin in the primary prevention of CVD in a proposed stepwise approach [129], effective and safe platelet-targeted strategies to prevent cardiovascular events in this clinical setting require further investigation. Another effect of aspirin is the acetylation of proteins other than cyclooxygenase, such as fibrinogen [130]. This leads to increased fibrin clot fiber thickness, increased clot permeability, and decreased clot lysis time [131, 132]. Mass-spectrometry analysis of fibrinogen incubated with aspirin identified 10 sites of acetylation but did not find any evidence for competition between glycation and acetylation [133]. A mass spectrometric study of plasma-based clots from T2DM patients has demonstrated that the intensity of fibrinogen acetylation, as well as clot properties, were unaffected by aspirin in a dose of 75 mg taken once daily [104]. However, this study also showed that glycation may block sites which were previously identified in vitro to be acetylated on fibrinogen [104]. Possible interference between these two processes has recently been suggested for two lysine residues on α2-antiplasmin [106], with a potential reversal of glycation by aspirin [107].

Results from the Cardiovascular Outcomes for People Using Anticoagulation Strategies (COMPASS) study, specifically a sub-study in diabetic patients by Bhatt et al., are in line with the antithrombotic effects of aspirin targeting clot properties [134]. This study demonstrated that the addition of rivaroxaban to aspirin resulted in a 26% decreased hazard ratio of cardiovascular events at the cost of increased risk of major bleeding [134]. These results were consistent with another trial, carried out in patients with unstable CHD, including 30% with diabetes, that demonstrated improvement in ischemic outcomes by addition of low-dose rivaroxaban (2.5 mg bid) on top of dual anti-platelet therapy [135]. The benefits of additional therapy with low-dose rivaroxaban are predicted to be even greater in real-word practice than in clinical trials [136]. The current evidence regarding the effects of rivaroxaban on clot properties comes largely from studies performed in nondiabetic settings. In a plasma-based study, treatment with rivaroxaban 20 mg once daily resulted in a 25% decrease in clot lysis time compared to baseline after 2–6 h from administration, with a return of clot lysis time to baseline after 20–25 h from administration [137]. In a cohort of atrial fibrillation patients, including 18% with diabetes, apixaban has been demonstrated to enhance endogenous fibrinolysis measured in the Global Thrombosis Test, with maximal effects in those with impaired fibrinolysis pre-treatment [138]. The effect of rivaroxaban 2.5 mg bid on clot properties and fibrinolysis in T2DM patients remains to be better characterized. If the fibrinolysis tests could be helpful in identifying the T2DM patients who should receive such therapy, this requires substantial research efforts to reach the following goals: characterization of the effect of low-dose FXa inhibitors on fibrinolysis in human plasma; identification of the clinical and biochemical determinants of the well and poor response to low-dose FXa inhibitors; and implementation of the global standard of testing fibrinolysis in T2DM patients by diabetologists and cardiologists before introducing anticoagulant drugs. Taken together, the modulation of fibrinolysis could be an important element of therapy in diabetic patients with the goal of reducing mortality, which is closely associated with cardiovascular disease. Pharmacological factors affecting fibrinolysis in T2DM have been summarized in Table 2.

## Conclusions

The current evidence indicates that impaired fibrinolysis characterizes patients with T2DM. However, the optimal test to assess the efficiency of fibrinolysis in this disease remains to be established given several assays used in clinical studies in recent years and their limitations. Recent studies have identified novel mechanisms involved in hypofibrinolysis in T2DM, including NETs and posttranslational modifications of proteins involved in fibrin formation and lysis. Over the past decade, several unexpected findings were revealed, including the fact that prolonged T2DM duration and decreased glycemic levels are factors contributing to altered fibrin structure and impaired lysis. The beneficial effects of antidiabetic drugs, in particular metformin, could be at least in part associated with fibrinolysis. Novel antidiabetic drugs are undoubtedly an integral part of T2DM treatment. Because of this, it is required that further studies regarding their impact on fibrinolysis be conducted. Due to the major involvement of protein glycation, it might be speculated that all hypoglycemic agents may improve fibrinolysis. In addition, treatment with low-dose FXa inhibitors and aspirin in diabetic patients at high risk for cardiovascular mortality could also be beneficial in terms of fibrinolysis. Presumably, multiple interventions are needed to improve fibrinolysis in T2DM. Further studies are needed to evaluate the actual role of enhanced fibrinolysis in the prevention of morbidity and mortality in a rapidly growing population of patients with T2DM worldwide.

## Data Availability

Not applicable.

## Abbreviations

8-iso-PGF_2α_: 8-iso-prostaglandin F_2α_
AGE: Advanced glycation end-products
BMI: Body mass index
CAT: Calibrated automated thrombogram
CHD: Coronary heart disease
CRP: C-reactive protein
CVD: Cardiovascular disease
DM: Diabetes mellitus
DPP-4: Dipeptidyl peptidase-4
DVT: Deep vein thrombosis
FXIII: Factor XIII
HbA1c: Glycated hemoglobin
IL-6: Interleukin 6
MI: Myocardial infarction
NETs: Neutrophil extracellular traps
NO: Nitric oxide
NOS: Nitric oxide synthase
NOX: NADPH oxidases
PAI-1: Plasminogen activator inhibitor 1
PMP: Platelet-derived microparticles
ROS: Reactive oxygen species
sCD40L: Soluble CD40 ligand
T2DM: Type 2 diabetes mellitus
TAFI: Thrombin-activatable fibrinolysis inhibitor
TG: Thrombin generation
TF: Tissue factor
tPA: Tissue plasminogen activator
TX: Thromboxane
VTE: Venous thromboembolism

## References

[1] Saeedi P, Petersohn I, Salpea P, Malanda B, Karuranga S, Unwin N (2019). Global and regional diabetes prevalence estimates for 2019 and projections for 2030 and 2045: results from the International Diabetes Federation Diabetes Atlas, 9th edition. Diabetes Res Clin Pract.

[2] Einarson TR, Acs A, Ludwig C, Panton UH (2018). Prevalence of cardiovascular disease in type 2 diabetes: a systematic literature review of scientific evidence from across the world in 2007–2017. Cardiovasc Diabetol.

[3] McGurnaghan S, Blackbourn LAK, Mocevic E, Haagen Panton U, McCrimmon RJ, Sattar N (2019). Cardiovascular disease prevalence and risk factor prevalence in Type 2 diabetes: a contemporary analysis. Diabet Med.

[4] Szczerbiński Ł, Gościk J, Bauer W, Wawrusiewicz-Kurylonek N, Paczkowska-Abdulsalam M, Niemira M (2019). Efficacy of family history, genetic risk score, and physical activity in assessing the prevalence of type 2 diabetes. Pol Arch Intern Med.

[5] Rungby J, Schou M, Warrer P, Ytte L, Andersen GS (2017). Prevalence of cardiovascular disease and evaluation of standard of care in type 2 diabetes: a nationwide study in primary care. Cardiovasc Endocrinol.

[6] Virani SS, Alonso A, Benjamin EJ, Bittencourt MS, Callaway CW, Carson AP (2020). Heart disease and stroke statistics—2020 update: a report from the American Heart Association. Circulation.

[7] Avogaro A, Giorda C, Maggini M, Mannucci E, Raschetti R, Lombardo F (2007). Incidence of coronary heart disease in type 2 diabetic men and women: impact of microvascular complications, treatment, and geographic location. Diabetes Care.

[8] Sarwar N, Gao P, Kondapally Seshasai SR, Gobin R, Kaptoge S, The Emerging Risk Factors Collaboration (2010). Diabetes mellitus, fasting blood glucose concentration, and risk of vascular disease: a collaborative meta-analysis of 102 prospective studies. Lancet.

[9] Grant PJ (2007). Diabetes mellitus as a prothrombotic condition. J Intern Med.

[10] Parma Z, Young R, Roleder T, Marona M, Ford I, Tendera M (2019). Management strategies and 5-year outcomes in Polish patients with stable coronary artery disease versus other European countries: data from the CLARIFY registry. Pol Arch Intern Med.

[11] Heit JA (2015). Epidemiology of venous thromboembolism. Nat Rev Cardiol.

[12] Tsai AW, Cushman M, Rosamond WD, Heckbert SR, Polak JF, Folsom AR (2002). Cardiovascular risk factors and venous thromboembolism incidence: the longitudinal investigation of thromboembolism etiology. Arch Intern Med.

[13] Piazza G, Goldhaber SZ, Kroll A, Goldberg RJ, Emery C, Spencer FA (2012). Venous thromboembolism in patients with diabetes mellitus. Am J Med.

[14] Heit JA, Leibson CL, Ashrani AA, Petterson TM, Bailey KRB, Melton LJ (2009). Is diabetes mellitus an independent risk factor for venous thromboembolism? A population-based case–control study. Arterioscler Thromb Vasc Biol.

[15] Bell EJ, Folsom AR, Lutsey PL, Elizabeth S, Zakai NA, Cushman M (2016). Diabetes mellitus and venous thromboembolism: a systematic review and meta-analysis. Diabetes Res Clin Pr.

[16] Galanaud JP, Bertoletti L, Amitrano M, Fernández-Capitán C, Pedrajas JM, Rosa V (2018). Predictors of post-thrombotic ulcer after acute DVT: the RIETE registry. Thromb Haemost.

[17] Lisman T, Leebeek FW, Mosnier LO, Bouma BN, Meijers JC, Janssen HL (2001). Thrombin-activatable fibrinolysis inhibitor deficiency in cirrhosis is not associated with increased plasma fibrinolysis. Gastroenterology.

[18] Carter AM, Cymbalista CM, Spector TD, Grant PJ (2007). Heritability of clot formation, morphology, and lysis. The EuroCLOT study. Arter Thromb Vasc Biol.

[19] Pieters M, Undas A, Marchi R, De Maat MPM, Weisel JW, Ariëns RAS (2012). An international study on the standardization of fibrin clot permeability measurement: methodological considerations and implications for healthy control values. J Thromb Haemost.

[20] Chitlur M, Rivard GE, Lillicrap D, Mann K, Shima M, Young G (2014). Recommendations for performing thromboelastography/thromboelastometry in hemophilia: communication from the SSC of the ISTH. J Thromb Haemost.

[21] Dunn EJ, Philippou H, Ariëns RAS, Grant PJ (2006). Molecular mechanisms involved in the resistance of fibrin to clot lysis by plasmin in subjects with type 2 diabetes mellitus. Diabetologia.

[22] Pieters M, Covic N, Loots DT, van der Westhuizen FH, van Zyl DG, Rheeder P (2006). The effect of glycaemic control on fibrin network structure of type 2 diabetic subjects. Thromb Haemost.

[23] Konieczynska M, Fil K, Bazanek M, Undas A (2014). Prolonged duration of type 2 diabetes is associated with increased thrombin generation, prothrombotic fibrin clot phenotype and impaired fibrinolysis. Thromb Haemost.

[24] Alzahrani SH, Hess K, Price JF, Strachan M, Baxter PD, Cubbon R (2012). Gender-specific alterations in fibrin structure function in type 2 diabetes: associations with cardiometabolic and vascular markers. J Clin Endocrinol Metab.

[25] Greenhalgh KA, Strachan MW, Alzahrani S, Baxter PD, Standeven KF, Storey RF (2017). Bβarg448lys polymorphism is associated with altered fibrin clot structure and fibrinolysis in type 2 diabetes. Thromb Haemost.

[26] Bochenek M, Zalewski J, Sadowski J, Undas A (2013). Type 2 diabetes as a modifier of fibrin clot properties in patients with coronary artery disease. J Thromb Thrombolysis.

[27] Sumaya W, Wallentin L, James SK, Siegbahn A, Gabrysch K, Bertilsson M (2018). Fibrin clot properties independently predict adverse clinical outcome following acute coronary syndrome: a PLATO substudy. Eur Heart J.

[28] Sumaya W, Wallentin L, James SK, Siegbahn A, Gabrysch K, Himmelmann A (2020). Impaired fibrinolysis predicts adverse outcome in acute coronary syndrome patients with diabetes: a PLATO sub-study. Thromb Haemost.

[29] Bryk AH, Konieczyńska M, Polak M, Plicner D, Bochenek M, Undas A (2021). Plasma fibrin clot properties and cardiovascular mortality in patients with type 2 diabetes: a long-term follow-up study. Cardiovasc Diabetol.

[30] Meltzer ME, Lisman T, Doggen CJM, de Groot PG, Rosendaal FR (2008). Synergistic effects of hypofibrinolysis and genetic and acquired risk factors on the risk of a first venous thrombosis. PLoS Med.

[31] Longstaff C, Kolev K (2015). Basic mechanisms and regulation of fibrinolysis. J Thromb Haemost.

[32] Davi G, Catalano I, Averna M, Notarbatolo A, Strano A, Ciabattoni G (1990). Thromboxane biosynthesis and platelet function in type II diabetes mellitus. N Engl J Med.

[33] Davì G, Gresele P, Violi F, Basili S, Catalano M, Giammarresi C (1997). Diabetes mellitus, hypercholesterolemia, and hypertension but not vascular disease per se are associated with persistent platelet activation in vivo: evidence derived from the study of peripheral arterial disease. Circulation.

[34] Davì G, Ciabattoni G, Consoli A, Mezzetti A, Falco A, Santarone S (1999). In vivo formation of 8-iso-prostaglandin and platelet activation in diabetes mellitus: effects of improved metabolic control and vitamin E supplementation. Circulation.

[35] Santilli F, Formoso G, Sbraccia P, Averna M, Miccoli R, Di Fulvio P (2010). Postprandial hyperglycemia is a determinant of platelet activation in early type 2 diabetes mellitus. J Thromb Haemost.

[36] Santilli F, Davì G, Consoli A, Cipollone F, Mezzetti A, Falco A (2006). Thromboxane-dependent CD40 ligand release in type 2 diabetes mellitus. J Am Coll Cardiol.

[37] Simeone P, Liani R, Tripaldi R, Di Castelnuovo A, Guagnano MT, Tartaro A (2018). Thromboxane-dependent platelet activation in obese subjects with prediabetes or early type 2 diabetes: effects of liraglutide-or lifestyle changes-induced weight loss. Nutrients.

[38] Santilli F, Lapenna D, La Barba S, Davì G (2015). Oxidative stress-related mechanisms affecting response to aspirin in diabetes mellitus. Free Radic Biol Med.

[39] Westerbacka J, Yki-Järvinen H, Turpeinen A, Rissanen A, Vehkavaara S, Syrjälä M (2002). Inhibition of platelet-collagen interaction. An in vivo action of insulin abolished by insulin resistance in obesity. Arter Thromb Vasc Biol.

[40] Schneider DJ, Hardison RM, Lopes N, Sobel BE, Brooks MM, Pro-Thrombosis Ancillary Study Group (2009). Association between increased platelet P-selectin expression and obesity in patients with type 2 diabetes. Diabetes Care.

[41] Altalhi R, Pechlivani N, Ajjan RA (2021). PAI-1 in diabetes: Pathophysiology and role as a therapeutic target. Int J Mol Sci.

[42] Torr-Brown SR, Sobel BE (1994). Plasminogen activator inhibitor is elevated in plasma and diminished in platelets in patients with diabetes mellitus. Thromb Res.

[43] Pretorius E, Oberholzer HM, Van Der Spuy WJ, Swanepoel AC, Soma P (2011). Qualitative scanning electron microscopy analysis of fibrin networks and platelet abnormalities in diabetes. Blood Coagul Fibrinolysis.

[44] Pretorius L, Thomson GJA, Adams RCM, Nell TA, Laubscher WA, Pretorius E (2018). Platelet activity and hypercoagulation in type 2 diabetes. Cardiovasc Diabetol.

[45] Giannella A, Ceolotto G, Radu CM, Cattelan A, Iori E, Benetti A (2021). PAR-4/Ca2+-calpain pathway activation stimulates platelet-derived microparticles in hyperglycemic type 2 diabetes. Cardiovasc Diabetol.

[46] Zubairova LD, Nabiullina RM, Nagaswami C, Zuev YF, Mustafin IG, Litvinov RI (2015). Circulating microparticles alter formation, structure, and properties of fibrin clots. Sci Rep.

[47] Soma P, Pretorius E (2015). Interplay between ultrastructural findings and atherothrombotic complications in type 2 diabetes mellitus. Cardiovasc Diabetol.

[48] Cines DB, Lebedeva T, Nagaswami C, Hayes V, Massefski W, Litvinov RI (2014). Clot contraction: compression of erythrocytes into tightly packed polyhedra and redistribution of platelets and fibrin. Blood.

[49] Gajos G, Siniarski A, Natorska J, Ząbczyk M, Siudut J, Malinowski KP (2018). Polyhedrocytes in blood clots of type 2 diabetic patients with high cardiovascular risk : association with glycemia, oxidative stress and platelet activation. Cardiovasc Diabetol.

[50] Jansson PA (2007). Endothelial dysfunction in insulin resistance and type 2 diabetes. J Intern Med.

[51] Hsueh WA, Law RE (1999). Insulin signaling in the arterial wall. Am J Cardiol.

[52] Aberer F, Pferschy PN, Tripolt NJ, Sourij C, Obermayer AM, Prüller F (2020). Hypoglycaemia leads to a delayed increase in platelet and coagulation activation markers in people with type 2 diabetes treated with metformin only: results from a stepwise hypoglycaemic clamp study. Diabetes Obes Metab.

[53] Joy NG, Mikeladze M, Younk LM, Tate DB, Davis SN (2016). Effects of equivalent sympathetic activation during hypoglycemia on endothelial function and pro-atherothrombotic balance in healthy individuals and obese standard treated type 2 diabetes. Metabolism.

[54] Schneider DJ, Absher PM, Ricci MA (1997). Dependence of augmentation of arterial endothelial cell expression of plasminogen activator inhibitor type 1 by insulin on soluble factors released from vascular smooth muscle cells. Circulation.

[55] Kooistra T, Bosma PJ, Töns HA, van den Berg AP, Meyer P, Princen HM (1989). Plasminogen activator inhibitor 1: biosynthesis and mRNA level are increased by insulin in cultured human hepatocytes. Thromb Haemost.

[56] Alessi MC, Peiretti F, Morange P, Henry M, Nalbone G, Juhan-Vague I (1997). Production of plasminogen activator inhibitor 1 by human adipose tissue: possible link between visceral fat accumulation and vascular disease. Diabetes.

[57] Leurs PB, Stolk RP, Hamulyak K, Van Oerle R, Grobbee DE, Wolffenbuttel BHR (2002). Tissue factor pathway inhibitor and other endothelium-dependent hemostatic factors in elderly individuals with normal or impaired glucose tolerance and type 2 diabetes. Diabetes Care.

[58] Bryk AH, Prior SM, Plens K, Konieczynska M, Hohendorff J, Malecki MT (2019). Predictors of neutrophil extracellular traps markers in type 2 diabetes mellitus: associations with a prothrombotic state and hypofibrinolysis. Cardiovasc Diabetol.

[59] Hori Y, Gabazza EC, Yano Y, Katsuki A, Suzuki K, Adachi Y (2002). Insulin resistance is associated with increased circulating level of thrombin-activatable fibrinolysis inhibitor in type 2 diabetic patients. J Clin Endocrinol Metab.

[60] Aso Y, Fujiwara Y, Tayama K, Takebayashi K, Inukai T, Takemura Y (2000). Relationship between soluble thrombomodulin in plasma and coagulation or fibrinolysis in type 2 diabetes. Clin Chim Acta.

[61] Meza CA, La Favor JD, Kim DH, Hickner RC (2019). Endothelial dysfunction: is there a hyperglycemia-induced imbalance of NOX and NOS?. Int J Mol Sci.

[62] Wautier MP, Chappey O, Corda S, Stern DM, Schmidt AM, Wautier JL (2001). Activation of NADPH oxidase by AGE links oxidant stress to altered gene expression via RAGE. Am J Physiol Endocrinol Metab.

[63] Østerud B, Bjørklid E (2006). Sources of tissue factor. Semin Thromb Hemost.

[64] Khechai F, Ollivier V, Bridey F, Amar M, Hakim J, De Prost D (1997). Effect of advanced glycation end product-modified on tissue factor expression by monocytes: role of oxidant stress and protein tyrosine kinase activation. Arterioscler Thromb Vasc Biol.

[65] Ichikawa K, Yoshinari M, Iwase M, Wakisaka M, Doi Y, Iino K (1998). Advanced glycosylation end products induced tissue factor expression in human monocyte-like U937 cells and increased tissue factor expression in monocytes from diabetic patients. Atherosclerosis.

[66] Boden G, Vaidyula VR, Homko C, Cheung P, Rao AK (2007). Circulating tissue factor procoagulant activity and thrombin generation in patients with type 2 diabetes: effects of insulin and glucose. J Clin Endocrinol Metab.

[67] Witkowski M, Tabaraie T, Steffens D, Friebel J, Dörner A, Skurk C (2018). MicroRNA-19a contributes to the epigenetic regulation of tissue factor in diabetes. Cardiovasc Diabetol.

[68] Witkowski M, Witkowski M, Saffarzadeh M, Friebel J, Tabaraie T, Ta Bao L (2020). Vascular miR-181b controls tissue factor-dependent thrombogenicity and inflammation in type 2 diabetes. Cardiovasc Diabetol.

[69] Wolberg AS, Campbell RA (2008). Thrombin generation, fibrin clot formation and hemostasis. Transfus Apher Sci.

[70] Tripodi A, Branchi A, Chantarangkul V, Clerici M, Merati G, Artoni A (2011). Hypercoagulability in patients with type 2 diabetes mellitus detected by a thrombin generation assay. J Thromb Thrombolysis.

[71] Ay L, Hoellerl F, Ay C, Brix JM, Koder S, Schernthaner GH (2012). Thrombin generation in type 2 diabetes with albuminuria and macrovascular disease. Eur J Clin Invest.

[72] Kim HK, Kim JE, Park SH, Kim YI, Nam-Goong IS, Kim ES (2014). High coagulation factor levels and low protein C levels contribute to enhanced thrombin generation in patients with diabetes who do not have macrovascular complications. J Diabetes Complicat.

[73] Frankel DS, Meigs JB, Massaro JM, Wilson PWF, O’Donnell CJ, D’Agostino RB (2008). Von Willebrand factor, type 2 diabetes mellitus, and risk of cardiovascular disease. The Framingham offspring study. Circulation.

[74] Beijers HJBH, Ferreira I, Spronk HMH, Bravenboer B, Dekker JM, Nijpels G (2012). Impaired glucose metabolism and type 2 diabetes are associated with hypercoagulability: potential role of central adiposity and low-grade inflammation—the Hoorn study. Thromb Res.

[75] Gajos G, Konieczynska M, Zalewski J, Undas A (2015). Low fasting glucose is associated with enhanced thrombin generation and unfavorable fibrin clot properties in type 2 diabetic patients with high cardiovascular risk. Cardiovasc Diabetol.

[76] Deeveraj S, Dasu MR, Jialal IJ (2010). Diabetes is a proinflammatory state: a translational perspective. Expert Rev Endocrinol Metab.

[77] Fuchs TA, Brill A, Duerschmied D, Schatzberg D, Monestier M, Myers DD (2010). Extracellular DNA traps promote thrombosis. Proc Natl Acad Sci.

[78] Joshi MB, Lad A, Bharath Prasad AS, Balakrishnan A, Ramachandra L, Satyamoorthy K (2013). High glucose modulates IL-6 mediated immune homeostasis through impeding neutrophil extracellular trap formation. FEBS Lett.

[79] Brinkmann V, Reichard U, Goosmann C, Fauler B, Uhlemann Y, Weiss DS (2004). Neutrophil extracellular traps kill bacteria. Science.

[80] Semeraro F, Ammollo CT, Morrissey JH, Dale GL, Friese P, Esmon NL (2011). Extracellular histones promote thrombin generation through platelet-dependent mechanisms: involvement of platelet TLR2 and TLR4. Blood.

[81] Longstaff C, Varjú I, Sótonyi P, Szabó L, Krumrey M, Hoell A (2013). Mechanical stability and fibrinolytic resistance of clots containing fibrin, DNA, and histones. J Biol Chem.

[82] Menegazzo L, Ciciliot S, Poncina N, Mazzucato M, Persano M, Bonora B (2015). NETosis is induced by high glucose and associated with type 2 diabetes. Acta Diabetol.

[83] Qi H, Yang S, Zhang L (2017). Neutrophil extracellular traps and endothelial dysfunction in atherosclerosis and thrombosis. Front Immunol.

[84] Howes J-M, Richardson VR, Smith KA, Schroeder V, Somani R, Shore A (2012). Complement C3 is a novel plasma clot component with anti-fibrinolytic properties. Diabetes Vasc Dis Res.

[85] Hess K, Alzahrani SH, Price JF, Strachan MW, Oxley N, King R (2014). Hypofibrinolysis in type 2 diabetes: the role of the inflammatory pathway and complement C3. Diabetologia.

[86] Alzahrani SH, Ajjan RA (2010). Coagulation and fibrinolysis in diabetes. Diabetes Vasc Dis Res.

[87] Kim PY, Stewart RJ, Lipson SM, Nesheim ME (2007). The relative kinetics of clotting and lysis provide a biochemical rationale for the correlation between elevated fibrinogen and cardiovascular disease. J Thromb Haemost.

[88] Ryan EA, Mockros LF, Weisel JW, Lorand L (1999). Structural origins of fibrin clot rheology. Biophys J.

[89] Bryk AH, Siudut J, Broniatowska E, Bagoly Z, Baráth B, Katona É (2020). Sex-specific alteration to α2-antiplasmin incorporation in patients with type 2 diabetes. Thromb Res.

[90] Bruno G, Merletti F, Biggeri A, Bargero G, Ferrero S, Pagano G (2005). Fibrinogen and AER are major independent predictors of 11-year cardiovascular mortality in type 2 diabetes: the Casale Monferrato study. Diabetologia.

[91] Lowe G, Woodward M, Hillis G, Rumley A, Li Q, Harrap S (2014). Circulating inflammatory markers and the risk of vascular complications and mortality in people with type 2 diabetes and cardiovascular disease or risk factors: the advance study. Diabetes.

[92] Zhang L, Xu C, Liu J, Bai X, Li R, Wang L (2019). Baseline plasma fibrinogen is associated with haemoglobin A1c and 2-year major adverse cardiovascular events following percutaneous coronary intervention in patients with acute coronary syndrome: a single-centre, prospective cohort study. Cardiovasc Diabetol.

[93] Kengne AP, Czernichow S, Stamatakis E, Hamer M, Batty GD (2013). Fibrinogen and future cardiovascular disease in people with diabetes: aetiological associations and risk prediction using individual participant data from nine community-based prospective cohort studies. Diabetes Vasc Dis Res.

[94] Dunn EJ, Ariëns RAS, Grant PJ (2005). The influence of type 2 diabetes on fibrin structure and function. Diabetologia.

[95] Undas A, Wiek I, Stepien E, Zmudka K, Tracz W (2008). Hyperglycemia is associated with enhanced thrombin formation, platelet activation, and fibrin clot resistance to lysis in patients with acute coronary syndrome. Diabetes Care.

[96] Neergaard-Petersen S, Hvas A-M, Kristensen SD, Grove EL, Larsen SB, Phoenix F (2014). The influence of type 2 diabetes on fibrin clot properties in patients with coronary artery disease. Thromb Haemost.

[97] Pieters M, Covic N, van der Westhuizen FH, Nagaswami C, Baras Y, Toit Loots D (2008). Glycaemic control improves fibrin network characteristics in type 2 diabetes—a purified fibrinogen model. Thromb Haemost.

[98] Li W, Sigley J, Pieters M, Helms CC, Nagaswami C, Weisel JW (2016). Fibrin fiber stiffness is strongly affected by fiber diameter, but not by fibrinogen glycation. Biophys J.

[99] Bryk AH, Zettl K, Wiśniewski JR, Undas A (2021). Glycation and acetylation sites on fibrinogen in plasma fibrin clot of patients with type 2 diabetes: effects of low-dose acetylsalicylic acid. Thromb Res.

[100] Lados-Krupa A, Konieczynska M, Chmiel A, Undas A (2015). Increased oxidation as an additional mechanism underlying reduced clot permeability and impaired fibrinolysis in type 2 diabetes. J Diabetes Res.

[101] Bryk A, Konieczynska M, Rostoff P, Broniatowska E, Hohendorff J, Malecki M (2019). Plasma protein oxidation as a determinant of impaired fibrinolysis in type 2 diabetes. Thromb Haemost.

[102] Baker SR, Ariëns RAS, Topaz On (2018). Fibrin clot structure and function: a novel risk factor for arterial and venous thrombosis and thromboembolism. Cardiovascular thrombus.

[103] Yurina L, Vasilyeva A, Indeykina M, Bugrova A, Biryukova M, Kononikhin A (2019). Ozone-induced damage of fibrinogen molecules: identification of oxidation sites by high-resolution mass spectrometry. Free Radic Res.

[104] Svensson J, Bergman A-C, Adamson U, Blombäck M, Wallén H, Jörneskog G (2012). Acetylation and glycation of fibrinogen in vitro occur at specific lysine residues in a concentration dependent manner: a mass spectrometric and isotope labeling study. Biochem Biophys Res Commun.

[105] Ajjan RA, Gamlen T, Standeven KF, Mughal S, Hess K, Smith KA (2013). Diabetes is associated with posttranslational modifications in plasminogen resulting in reduced plasmin generation and enzyme-specific activity. Blood.

[106] Bryk AH, Cysewski D, Dadlez M, Undas A (2020). Identification of glycated and acetylated lysine residues in human α2-antiplasmin. Biochem Biophys Res Commun.

[107] Bryk AH, Satała D, Natorska J, Rąpała-Kozik M, Undas A (2020). Interaction of glycated and acetylated human α2-antiplasmin with fibrin clots. Blood Coagul Fibrinolysis.

[108] de Willige SU, Malfliet JJCM, Abdul S, Leebeek FWG, Rijken DC (2018). The level of circulating fibroblast activation protein correlates with incorporation of alpha-2-antiplasmin into the fibrin clot. Thromb Res.

[109] Sobczak AIS, Stewart AJ (2019). Coagulatory defects in type-1 and type-2 diabetes. Int J Mol Sci.

[110] Lipinski B, Pretorius E (2013). Iron-induced fibrin in cardiovascular disease. Curr Neurovasc Res.

[111] Thiele L, Rau M, Hartmann N-UK, Moellmann J, Jankowski J, Boehm M (2021). Effects of empagliflozin on erythropoiesis in patients with type 2 diabetes—data from a randomized, placebo controlled study. Diabetes Obes Metab.

[112] UK Prospective Diabetes Study (UKPDS) (1998). Effect of intensive blood-glucose control with metformin on complications in overweight patients with type 2 diabetes (UKPDS 34). Lancet.

[113] Johnson JA, Majumdar SR, Simpson SH, Toth EL (2002). Decreased mortality associated with the use of metformin compared with sulfonylurea monotherapy in type 2 diabetes. Diabetes Care.

[114] Grant PJ (2003). Beneficial effects of metformin on haemostasis and vascular function in man. Diabetes Metab.

[115] Kender Z, Fleming T, Kopf S, Torzsa P, Grolmusz V, Herzig S (2014). Effect of metformin on methylglyoxal metabolism in patients with type 2 diabetes. Exp Clin Endocrinol Diabetes.

[116] Huang W, Xin G, Wei Z, Ji C, Zheng H, Gu J (2016). Metformin uniquely prevents thrombosis by inhibiting platelet activation and mtDNA release. Sci Rep.

[117] Menegazzo L, Scattolini V, Cappellari R, Bonora BM, Albiero M, Bortolozzi M (2018). The antidiabetic drug metformin blunts NETosis in vitro and reduces circulating NETosis biomarkers in vivo. Acta Diabetol.

[118] De Jager J, Kooy A, Lehert P, Bets D, Wulffelé MG, Teerlink T (2005). Effects of short-term treatment with metformin on markers of endothelial function and inflammatory activity in type 2 diabetes mellitus: a randomized, placebo-controlled trial. J Intern Med.

[119] Witkowski M, Friebel J, Tabaraie T, Grabitz S, Dörner A, Taghipour L (2021). Metformin is associated with reduced tissue factor procoagulant activity in patients with poorly controlled diabetes. Cardiovasc Drugs Ther.

[120] Standeven KF, Ariens RAS, Whitaker P, Ashcroft AE, Weisel JW, Grant PJ (2002). The effect of dimethylbiguanide on thrombin activity, FXIII activation, fibrin polymerization, and fibrin clot formation. Diabetes.

[121] Ishibashi Y, Matsui T, Maeda S, Higashimoto Y, Yamagishi S (2013). Advanced glycation end products evoke endothelial cell damage by stimulating soluble dipeptidyl peptidase-4 production and its interaction with mannose 6-phosphate/insulin-like growth factor II receptor. Cardiovasc Diabetol.

[122] Tani S, Takahashi A, Nagao K, Hirayama A (2015). Effect of dipeptidyl peptidase-4 inhibitor, vildagliptin on plasminogen activator inhibitor-1 in patients with diabetes mellitus. Am J Cardiol.

[123] Forst T, Anastassiadis E, Diessel S, Löffler A, Pfützner A (2014). Effect of linagliptin compared with glimepiride on postprandial glucose metabolism, islet cell function and vascular function parameters in patients with type 2 diabetes mellitus receiving ongoing metformin treatment. Diabetes Metab Res Rev.

[124] Bray JJH, Foster-Davies H, Salem A, Hoole AL, Obaid DR, Halcox JPJ (2021). Glucagon-like peptide-1 receptor Agonists (GLP-1RAs) improve biomarkers of inflammation and oxidative stress: a systematic review and meta-analysis of randomised controlled trials. Diabetes Obes Metab.

[125] Sakurai S, Jojima T, Iijima T, Tomaru T, Usui I, Aso Y (2020). Empagliflozin decreases the plasma concentration of plasminogen activator inhibitor-1 (PAI-1) in patients with type 2 diabetes: Association with improvement of fibrinolysis. J Diabetes Complicat.

[126] Rocca B, Santilli F, Pitocco D, Mucci L, Petrucci G, Vitacolonna E (2012). The recovery of platelet cyclooxygenase activity explains interindividual variability in responsiveness to low-dose aspirin in patients with and without diabetes. J Thromb Haemost.

[127] Rocca B, Patrono C (2020). Aspirin in the primary prevention of cardiovascular disease in diabetes mellitus: a new perspective. Diabetes Res Clin Pract.

[128] Khan SU, Ul Abideen Asad Z, Khan MU, Talluri S, Ali F, Khan MS (2020). Aspirin for primary prevention of cardiovascular outcomes in diabetes mellitus: an updated systematic review and meta-analysis. Eur J Prev Cardiol.

[129] Aimo A, De Caterina R (2020). Aspirin for primary cardiovascular prevention: why the wonder drug should not be precipitously dismissed. Pol Arch Intern Med.

[130] Bjornsson TD, Schneider DE, Berger H (1989). Aspirin acetylates fibrinogen and enhances fibrinolysis. Fibrinolytic effect is independent of changes in plasminogen activator levels. J Pharmacol Exp Ther.

[131] Undas A, Brummel-Ziedins KE, Mann KG (2014). Why does aspirin decrease the risk of venous thromboembolism? On old and novel antithrombotic effects of acetyl salicylic acid. J Thromb Haemost.

[132] Ajjan RA, Standeven KF, Khanbhai M, Phoenix F, Gersh KC, Weisel JW (2009). Effects of aspirin on clot structure and fibrinolysis using a novel in vitro cellular system. Arterioscler Thromb Vasc Biol.

[133] Svensson E, Baggesen LM, Johnsen SP, Pedersen L, Nørrelund H, Buhl ES (2017). Early glycemic control and magnitude of HbA1c reduction predict cardiovascular events and mortality: population-based cohort study of 24,752 metformin initiators. Diabetes Care.

[134] Bhatt DL, Eikelboom JW, Connolly SJ, Steg PG, Anand SS, Verma S (2020). Role of combination antiplatelet and anticoagulation therapy in diabetes mellitus and cardiovascular disease: insights from the COMPASS trial. Circulation.

[135] Mega J, Braunwald E, Wiviott S, Bassand J, Bhatt D, Bode C (2012). Rivaroxaban in patients with a recent acute coronary syndrome. N Engl J Med.

[136] Desperak P, Hudzik B, Gąsior M (2019). Assessment of patients with coronary artery disease who may benefit from the use of rivaroxaban in the real world: implementation of the COMPASS trial criteria in the TERCET registry population. Pol Arch Intern Med.

[137] Janion-Sadowska A, Natorska J, Siudut J, Zabczyk M, Stanisz A, Undas A (2017). Plasma fibrin clot properties in the G20210A prothrombin mutation carriers following venous thromboembolism: the effect of rivaroxaban. Thromb Haemost.

[138] Spinthakis N, Gue Y, Farag M, Srinivasan M, Wellsted D, Arachchillage DRJ (2019). Apixaban enhances endogenous fibrinolysis in patients with atrial fibrillation. Europace.

[139] Meltzer ME, Doggen CJM, de Groot PG, Rosendaal FR, Lisman T (2009). Reduced plasma fibrinolytic capacity as a potential risk factor for a first myocardial infarction in young men. Br J Haematol.

[140] Grant PJ (1996). The effects of high- and medium-dose metfformin therapy on cardiovascular risk factors in patients with type II diabetes. Diabetes Care.

